# The dual functional role of MicroRNA-18a (miR-18a) in cancer development

**DOI:** 10.1186/s40169-019-0250-9

**Published:** 2019-12-23

**Authors:** Kexin Shen, Zhe Cao, Ruizhe Zhu, Lei You, Taiping Zhang

**Affiliations:** 1 Department of General Surgery, Peking Union Medical College Hospital, Chinese Academy of Medical Sciences and Peking Union Medical College, Beijing, 100730 China; 2 Tsinghua University School of Medicine, Beijing, 100084 China

**Keywords:** MiR-18a, Biogenesis, Tumorigenesis, Oncogenesis, EMT, Clinical applications

## Abstract

The polycistronic miR-17-92 cluster is instrumental in physiological processes commonly dysregulated in cancer, such as proliferation, the cell cycle, apoptosis, and differentiation. MicroRNA-18a (miR-18a) is one of the most conserved and multifunctional miRNAs in the cluster and is frequently overexpressed in malignant tumors. Altered miR-18a expression has been found in various physiological and pathological processes, including cell proliferation, apoptosis, epithelial–mesenchymal transition (EMT), tumorigenesis, cancer invasion and metastasis. In this review, we summarized the molecular basis and regulatory targets of miR-18a in cancer development. Interestingly, miR-18a has a dual functional role in either promoting or inhibiting oncogenesis in different human cancers. The differential miRNA expression in cancers of the same organ at different stages or of various subtypes suggests that this dual function of miR-18a is independent of cancer type and may be attributed to the fundamental differences in tumorigenic mechanisms. Finally, we summarized the current clinical use of miR-18a and discussed its potential uses in cancer therapy.

## Background

MicroRNAs (miRNAs) are small noncoding RNAs of 19–22 nucleotides in length that play a critical role in preventing the translation of target proteins by degrading or binding to the 3′ untranslated region (3′-UTR) of the corresponding messenger RNAs (mRNAs) [1]. The first miRNA was discovered in *Caenorhabditis elegans* (lin-4 locus2) in 1993 [2], and the first miRNA in mammals (let-7) was discovered in 2000 [3]. These two studies provided the foundation for subsequent recent findings revealing that miRNAs are involved in almost all essential biological processes, including cell proliferation, differentiation, and apoptosis [4–10]. The earliest discovered miRNAs listed above are named after their associated phenotypes, whereas the names of the lin-4 homologs in other species are designated numerically by the order of their discovery as miR‐125. Genes encoding sister miRNAs are indicated with the suffix letters a and b, and each locus produces miRNAs from both the 5ʹ strand and the 3ʹ strand of the precursor, respectively [8].

**Fig. 1:**
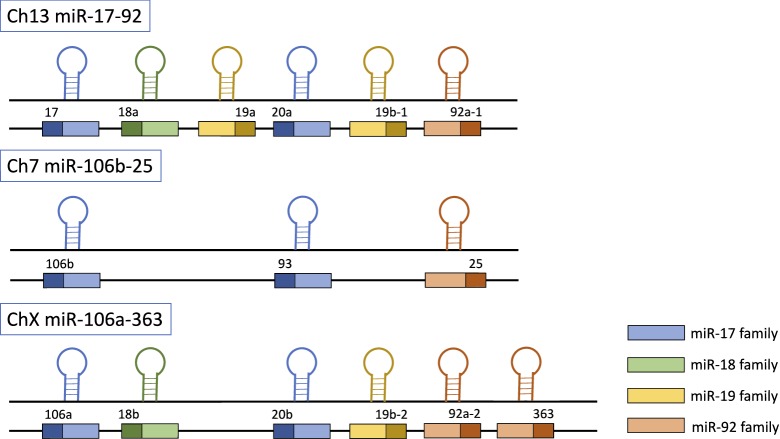
The human miR-17-92 gene and its homologs. The gene structure of the human miR-17-92 cluster (located on chromosome 13) and its two mammalian paralogs: the miR-106b-25 cluster (located on human chromosome 7) and the miR-106a-363 cluster (located on the X chromosome). The miRNAs encoded by the 3 clusters are categorized into the miR-17 family (miR-17, miR-20a, miR-106b, miR-93, miR-106a and miR-20b), the miR-18a family (miR-18a and miR-18b), the miR-19 family (miR-19a, miR-19b-1 and miR-19b-2) and the miR-92 family (miR-92a-1, miR-25, miR-92a-2 and miR-363)

The polycistronic miR-17-92 cluster is located in the 13q31.3 region of human chromosome 13 and produces seven individual mature miRNAs: miR-17-3p, miR-17-5p, miR-18a, miR-19a, miR-20a, miR-19b, and miR-92a [11–14]. This cluster has two mammalian paralogs: the miR-106b-25 cluster (located on human chromosome 7) and the miR-106a-363 cluster (located on the X chromosome). The miR-17-92 gene cluster and its homologous genes can be divided into four families based on their seed sequences: the miR-17 family (miR-17, miR-20a/miR-20b, miR-106a/miR-106b, and miR-93), the miR-18 family (miR-18a/miR-18b), the miR-19 family (miR-19a/miR-19b), and the miR-25 family (miR-25, miR-92a, and miR-363) [15] (Fig. 1). Members of the miRNA gene cluster cooperate in the regulation of certain processes or play an associated role in the same biological process, ensuring orderly and more efficient biological activity [16]. The interaction of miRNAs with the 3′-UTR of mRNAs depends on a 7-nucleotide sequence at the 5′ end (nucleotides 2–8) of the miRNA. The results of many bioinformatic and experimental analyses have indicated that miRNAs regulate protein-coding genes [17–19].

The importance of miRNAs is emphasized in both physical and pathological states. Over the past decade, cancer has been the major focus of many studies about miRNA function. Dysregulation of miRNA expression is associated with many cancers [20]. Some studies have demonstrated that miRNAs exert important effects by controlling the expression of their target mRNAs to facilitate cancer cell proliferation, epithelial–mesenchymal transition (EMT) [10], and cancer metastasis [21]. In addition, tumor miRNA profiles can provide information about drug resistance [22]. Finally, tumor miRNAs can be detected in biological fluids, thus acting as biomarkers for tumor screening and monitoring for certain neoplastic diseases [23]. This review focuses on miR-18a due to its unique conserved sequence, biogenesis, regulation, dual function in several cancers and potential as a biomarker in human cancer screening.

## Main text

### Exosomal miR-18a biogenesis

The biogenesis of miRNAs is strictly regulated at multiple levels, including transcription by RNA polymerase II (Pol II), maturation by RNase III proteins, posttranscriptional modifications, Argonaute (AGO) family protein loading and RNA decay [8].

Pol II is regulated by RNA Pol II-associated transcription factors, such as p53, MYC, ZEB1, ZEB2, and myoblast determination protein 1 (MYOD1) [24, 25]. p53, MYC, and MYOD1 transactivate the miR-34, miR‐17 and miR‐1 cluster, respectively. MYC transcriptionally suppresses the miR‐15a cluster, and ZEB1 and ZEB2 transcriptionally suppress the miR-200 cluster. Epigenetic regulation, including histone modification and DNA methylation, also contributes to the miRNA transcription process. The primary miRNA (pri-miRNA) undergoes maturation initiated by the nuclear RNase III Drosha via cropping of the stem-loop into a small hairpin-shaped RNA molecule of ~ 65 nucleotides (called the pre-miRNA) [26]. As cleavage by Drosha defines the terminus and therefore the specificity of the miRNA, Microprocessor, a complex formed by Drosha with its cofactor DiGeorge syndrome chromosomal (or critical) region 8 (DGCR8), controls the recognition of pri-miRNA processing [27]. This process is achieved by an autoregulatory mechanism in which DGCR8 stabilizes Drosha while Drosha destabilizes DGCR8 [28–31]. Next, the pre-miRNA is exported into the cytoplasm by exportin 5 (EXP 5). However, some noncanonical pre-miRNAs (for example, pre-mir-320) are directly generated through transcription—bypassing Drosha processing—and are exported into the cytoplasm by exportin 1 (EXP 1) [32].

The exported cytoplasmic pre-miRNA is cleaved by Dicer near the terminal loop, liberating a small RNA duplex. Dicer interacts with a double-stranded RNA-binding domain (dsRBD) protein called transactivation response (TAR) RNA-binding protein (TRBP) in humans and Loquacious (Loqs) in flies [33–35]. Following processing by Dicer, the RNA duplex is released and subsequently loaded onto the human AGO1–4 proteins to form an effector complex called the RNA-induced silencing complex (RISC) [36, 37]. In the miRISC-related pathway, a complex formed by heat shock cognate 70 (HSC70) and heat shock protein 90 (HSP90) hydrolyzes ATP to load the RNA duplex [38]. The mature strand remains in one of the AGO proteins, and the other strand is discarded. These mature miRNAs are translocated into the recipient cells directly by microvesicles or are loaded into multivesicular bodies (MVBs), which dock onto the cell membrane and release exosomes into the extracellular fluid (ECF). Exosome fusion with recipient cells or phagocytosis leads to the release of miRNA into the cytosol and, in turn, to translational repression.

Exosomes are nanovesicles that carry proteins, mRNAs, and miRNAs involved in intercellular communication and the regulation of different biological processes through the circulatory system. These vesicles can be transferred from donor to recipient cells via fusion to the target cell membrane and have recently been recognized as essential mediators of intercellular interactions. Exosomal miRNAs are potentially present in the tumor microenvironment [39], because exosome transfer is indispensable for the distribution of cancer-promoting cellular contents to the surrounding cells, which accelerates cancer progression [40]. Understanding the transfer of exosomal miRNAs to recipient cells to regulate target gene expression during this process is important to both understanding the biological mechanism underlying cancer progression and further exploring therapeutic approaches [41]. Although limited research has been performed on exosomal miR-18a, its more common form—circulating miR-18a—will be further discussed in section “MiR-18a in clinical applications”.

Although Microprocessor (the Drosha-DGCR8 complex) is necessary and sufficient for the processing of some miRNAs, the biogenesis of a subset of miRNAs is regulated by additional factors. Recent evidence suggests that several positive and negative trans-acting factors regulate the canonical miRNA processing pathway. For instance, heterogeneous nuclear ribonucleoprotein A1 (hnRNP A1) is an RNA-binding protein that positively regulates the biogenesis of miR-18a [42]. This multifunctional RNA-binding protein is required for the processing of miR-18a at the nuclear step of Drosha-mediated processing [43]. Structural and functional analysis of RNA showed that hnRNP A1 regulates pri-miR-18a processing by binding to the terminal loop of pri-miR-18a and altering its stem-loop structure, enhancing the effectiveness of Drosha-mediated cleavage.

**Fig. 2:**
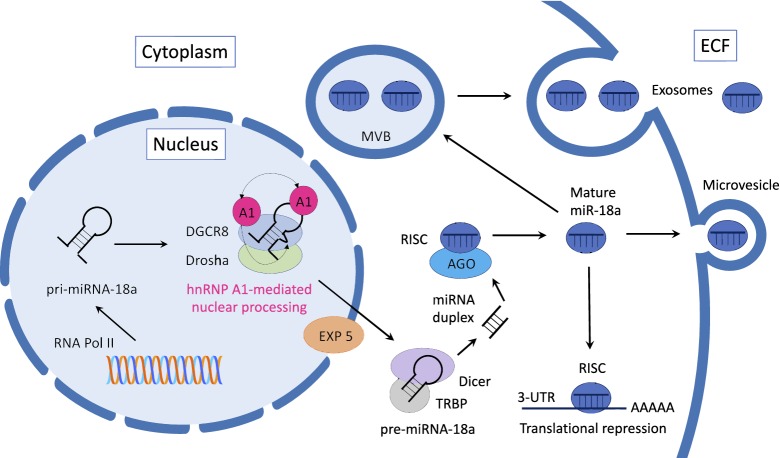
The biogenesis of miR-18a. Model of miR-18a biogenesis, function and secretion. The miR-18a gene is transcribed into pri-miR-18a in the nucleus and is processed by the Microprocessor complex, which is composed of Drosha and DGCR8, to generate pre-miR-18a. Pre-miR-18a is then exported into the cytoplasm by exportin 5 (EXP 5) and cleaved by Dicer into a miRNA duplex. One strand of the duplex is selectively loaded into the RNA-induced silencing complex (RISC) along with argonaut (AGO). Mature miR-18a in the RISC complex can engage in base pairing with its target mRNA and induce translational repression or can be transferred into recipient cells directly by microvesicles or loaded into multivesicular bodies (MVBs) and exosomes, which are subsequently secreted into the extracellular space

Furthermore, the binding of hnRNP A1 to pri-miR-18a is linked to a unique conserved sequence in its terminal loop [44]. Bioinformatic and mutational analyses revealed that some pri-miRNAs have highly conserved terminal loops that act as “landing pads” for trans-acting factors that influence miRNA processing. Figure 2 shows a model of the mechanism by which hnRNP A1 facilitates Drosha-mediated processing of pri-miR-18a.

As discussed previously, the processing of pro-miR-18a to pre-miR-18a is mediated by Microprocessor in cooperation with multiple factors, including hnRNP A1. In the biogenesis step upstream of the regulatory effects of Microprocessor, pri-miR-18a adopts an RNA conformation that selectively blocks Microprocessor. Pri-miR-18a is processed to the intermediate pro-miRNA form, which is an efficient substrate for Microprocessor and is required to selectively allow the production of pre-miR-18a [45–50].

A study of embryonic stem cells (ESCs) during differentiation described a new paradigm for pri-miR-18a regulation in which specific repression domain (RD) sequences participate in the formation of a higher-order RNA conformation that selectively inhibits Microprocessor-mediated production of pre-miR-18a [51]. This process provides an additional processing step upstream of Microprocessor that could be dynamically regulated for precise miRNA control. The RD in the 5′ region of pri-miR-17-92 was confirmed to selectively inhibit the production of most miRNAs in the cluster, including miR-18a, but does not inhibit the processing of pre-miR-92; this autoinhibitory mechanism might explain the posttranscriptional regulation of pri-miR-18a expression.

In addition, the endonuclease cleavage and polyadenylation specificity factor subunit 3 (CPSF3) and the spliceosome-associated ISY1 splicing factor homolog (ISY1) control pro-miRNA biogenesis and expression [52, 53]. Unlike DGCR8, which associated with all tested RNAs, CPSF3 and ISY1 associated specifically with the cleavage site mutant of pri-miR-17-92. Both CPSF3 and ISY1 are selectively required for the expression of all miRNAs except miR-92 within the polycistronic miR-17-92 cluster, including miR-18a. In conclusion, the developmentally regulated generation of pro-miRNA-18a is a crucial step in the control of miRNA-18a expression and explains the posttranscriptional control of miR-18a expression in ESCs.

Recent studies have gradually established connections between miRNA dysregulation and cancer development. Interestingly, the functions of miR-18a not only in promoting the malignant progression of human cancers but also in inhibiting the proliferation of specific tumor cells as well as EMT associated with cancer metastasis have been demonstrated, as further discussed in the following section.

### The dual functional role of miR-18a in cancer development

Despite emerging evidence about the role of miRNAs in cancer progression, fewer studies have been published on the functions of miR-18a in cancer development than on the functions of other miRNAs in the miR-17-92 cluster. Most research has focused on specific targets of miR-18a that are involved in canonical pathways associated with cell proliferation, EMT, and the microenvironment. Interestingly, miR-18a has been found to either promote or inhibit tumorigenesis, suggesting its dual function in cancer progression. Here, we summarize the targets of miR-18a and their effects in different cancers (Table 1).

**Table 1:** List of experimentally validated miR-18a targets and their biological roles

Target gene	Full name	Verification methods	Cell lines	Biological roles	References
IRF2	Interferon regulatory factor 2	Luciferase reporter assay	BEAS-2B, A549, HEK-293T	Promotes apoptosis, inhibits cell proliferation and migration	[54]
SOX6	Sex-determining region Y (SRY)-box 6	Luciferase reporter assay, Western blotting	CaSki, ME-180, SiHa, PaCa-2	Inhibits the Wnt/β-catenin pathway	[56]
WNK2	WNK lysine deficient protein kinase 2	Luciferase reporter assay, Western blotting	CaSki, ME-180, SiHa, PaCa-2	Inhibits the MEK/ERK pathway	[56]
PTEN	Phosphatase and tensin homolog	Luciferase reporter assay, Western blotting	CaSki, ME180, SiHa, PaCa-2	Inhibits the PI3K/AKT pathway	[56]
STK4	Serine/threonine-protein kinase 4	Luciferase reporter assay, qRT-PCR	HEK-293T, DU145	Promotes apoptosis, inhibits endogenous AKT activity	[58]
PIAS3	Protein inhibitor of activated STAT3 (signal transducer and activator of transcription 3)	Immunoprecipitation assay, qRT-PCR	MKN28, MKN1	Inhibits STAT3 activity, downstream of the Wnt/β-catenin pathway	[61, 66]
CDC42	Cell division control protein 42	Luciferase reporter assay	HCT116	Acts as a GTPase in the PI3K/AKT pathway	[68]
SREBP1	Sterol regulatory element-binding transcription protein 1	Luciferase reporter assay, Transwell assay, qRT-PCR	MDA-MB-231, MCF-7	Promotes EMT and cell migration and invasion	[69]
CTGF	Connective tissue growth factor	Luciferase reporter assay, Western blotting, qRT-PCR	3T3	Promotes cell proliferation, phosphorylation of ERK1/2 and AKT, crosstalk with the TGF-β pathway	[75]
Nedd9	Neural precursor cell expressed, developmentally downregulated 9	Luciferase reporter assay, Western blotting, qRT-PCR	3T3	Downstream of the Wnt/β-catenin pathway, activates the MEK/ERK pathway	[75]

#### MiR-18a was shown to promote the malignant progression of lung cancer, cervical cancer (CC), prostate cancer, gastric cancer (GC), and mesothelioma by regulating downstream targets and inducing cancer development

A study in non-small-cell lung cancer (NSCLC) showed that miR-18 was significantly upregulated in both NSCLC tissues and cell lines, suggesting an oncogenic role of miR-18a in lung cancer [54]. This study focused on the mechanism of miR-18a and confirmed that miR-18a promoted cell proliferation by directly targeting interferon regulatory factor 2 (IRF2), an IRF family member that plays a crucial role in adaptive immunity and modulates cellular responses involved in tumorigenesis [55]. The 3′-UTR of IRF2 was verified by a luciferase assay as a target of miR-18a. Further experiments indicated that IRF2 enhanced apoptosis and inhibited cell proliferation and migration, suggesting that miR-18a can promote NSCLC development through IRF2 downregulation. Additionally, the study demonstrated that overexpression of miR-18a induced autophagy in a manner positively correlated with EGFR expression in vivo. This research group also noted a positive correlation between miR-18a and NFκB. However, they did not further investigate this regulatory process. Based on these results, this study provided new insights into the mechanism by which miR-18a contributes to oncogenesis in lung cancer, suggesting potential targets for future diagnostic strategies and treatments.

Based on the previously reported upregulation of miR-18a in CC samples, a study investigated the roles of miR-18a in mediating PD-L1 expression and CC progression [56]. PD-L1 is a crucial inhibitory immune receptor that is overexpressed in CC [57]. This study also demonstrated that depletion of PD-L1 suppresses the proliferation, invasion, and tumorigenesis of CC cells. Because phosphatase and tensin homolog (PTEN), WNK lysine deficient protein kinase 2 (WNK2), and sex-determining region Y (SRY)-box 6 (SOX6) have miR-18a-binding sites in their 3′-UTRs and act as tumor suppressors in CC, this study hypothesized and confirmed that miR-18a indirectly upregulates PD-L1 expression by activating the phosphatidylinositol 3-kinase-protein kinase B (PI3K/AKT), MEK/extracellular signal-regulated kinase (ERK), and Wnt/β-catenin pathways. Furthermore, SOX6 is an activator of the p53 pathway, and p53 suppresses PD-L1 expression, suggesting that SOX6 targeting by miR-18a also increased PD-L1 levels by inactivating p53 signaling. By identifying OCT4 binding sites, this research group not only found a positive correlation between OCT4 and miR-18a but also confirmed that OCT4 triggered miR-18a overexpression in CC cells. According to these findings, this study proposed the OCT4-miR-18a-PTEN/WNK2/SOX6 oncogenic axis as a mechanism contributing to PD-L1 overexpression in CC.

One study showed that the 3′-UTR of serine/threonine-protein kinase 4 (STK4) is a direct target of miR-18a, which is highly expressed in prostate cancer cell lines [58]. STK4 is a homolog of Hippo (Hpo/hpo) in Drosophila and was originally identified as a proapoptotic kinase and an inhibitor of endogenous AKT [59]. Overexpression of miR-18a in prostate cancer cells decreased STK4 protein expression and enhanced colony formation. This study further investigated the associations among miR-18a, STK4 and AKT and confirmed that miR-18a enhanced AKT phosphorylation by decreasing STK4 levels in prostate cancer cells. This finding was further supported by the results of separate experiments examining the expression of the downstream apoptosis marker poly-(ADP-ribose) polymerase (PARP) in response to antagonizing miR-18a or inducing STK4 expression. Both actions resulted in enhanced PARP cleavage, thereby inducing apoptosis. These data suggest that miR-18a expression is elevated in prostate cancer and promotes tumorigenesis both in vitro and in vivo by suppressing STK4. By targeting miR-18a, inhibiting the miR-18a/STK4 interaction or restoring STK4 expression, new miR-18a-related therapeutic strategies could be applied to future prostate cancer treatment.

In GC, mRNA-18a modulates signal transducer and activator of transcription 3 (STAT3) activity through negatively regulating protein inhibitor of activated STAT3 (PIAS3) [60]. In a study that used fluorescence in situ hybridization (FISH) and copy number analysis in gastric tissue microarray (TMA) specimens to examine miR-17-92 cluster expression, miR-18a expression was higher than that of other miRNAs in the cluster [61]. Activated STAT3 protects tumor cells from apoptosis and promotes cell proliferation by regulating genes encoding antiapoptotic and proliferation-associated proteins, such as Bcl-xL, survivin, and c-myc [62]. PIAS3 inhibits STAT3, and PIAS3 levels are negatively correlated with Bcl-xL, survivin, and c-myc levels. The study determined that miR-18a directly targets the 3′-UTR of PIAS3, leading to enhanced activation of genes downstream of STAT3. Bcl-xL is overexpressed at both the RNA and protein levels in GCs [63], c-myc is a potent inducer of proliferation [64], and activated survivin inhibits GC cell apoptosis [65]. The study concluded that miR-18a is an oncogene and plays a vital role in GC development by negatively regulating PIAS3 and thereby modulating STAT3 target genes.

The function of miR-18a as a suppressor of PIAS3 was also identified in malignant mesothelioma (MM) [66]. The referenced study screened candidate miRNAs in MM cell lines with low PIAS3 expression and conducted luciferase reporter assays to validate the regulation via the 3′-UTR of PIAS3. The researchers found that miR-18a suppressed PIAS3 expression and that miR-18a inhibition increased PIAS3 expression. Moreover, the study showed that miR-18a expression was negatively correlated with MM patient survival and that miR-18a inhibition decreased MM cell viability, suggesting that the effects of posttranscriptional regulation of miR-18a on PIAS3 expression also contribute to MM development. Another similar study demonstrated that miR-18a activated the IL-6/STAT3 signaling pathway by suppressing the expression of PIAS3 in human hepatocytes [67], suggesting that miR-18a also plays a role in the STAT3 signaling pathway during nontumorigenic events.

#### MiR-18a was shown to inhibit the malignant progression of colorectal cancer (CRC) and breast cancer (BC) and to inhibit the proliferation of pancreatic progenitor cells, suggesting that miR-18a has a dual functional role in cancer development (Fig. 3)

In a study of CRC, miR-18a was found to play a tumor suppressor role by inhibiting cell division control protein 42 (CDC42), a mediator of the PI3K pathway [68]. CDC42 expression is increased in multiple human cancers, including CRC [38], and CDC42 overexpression may also contribute to cancer therapeutic resistance [39]. Recent evidence has indicated that CDC42 activation is a crucial step in the malignant progression of colorectal cells [40]. This study focused on CDC42 regulation and found that miR-18a directly targeted the 3′-UTR of CDC42 to reduce CDC42 expression. Preventing the binding of miR-18a with target protectors confirmed that the direct targeting of CDC42 by miR-18a is indispensable for the reduction in cell growth observed at high miR-18a levels. Moreover, knockdown of CDC42 activated p53, leading to apoptosis; in contrast, blocking CDC42 mRNA from suppression by miR-18a restored cell growth, suggesting that miR-18a exerts its tumor suppressor role in CRC via the PI3K pathway by targeting CDC42 and reducing its expression.

**Fig. 3:**
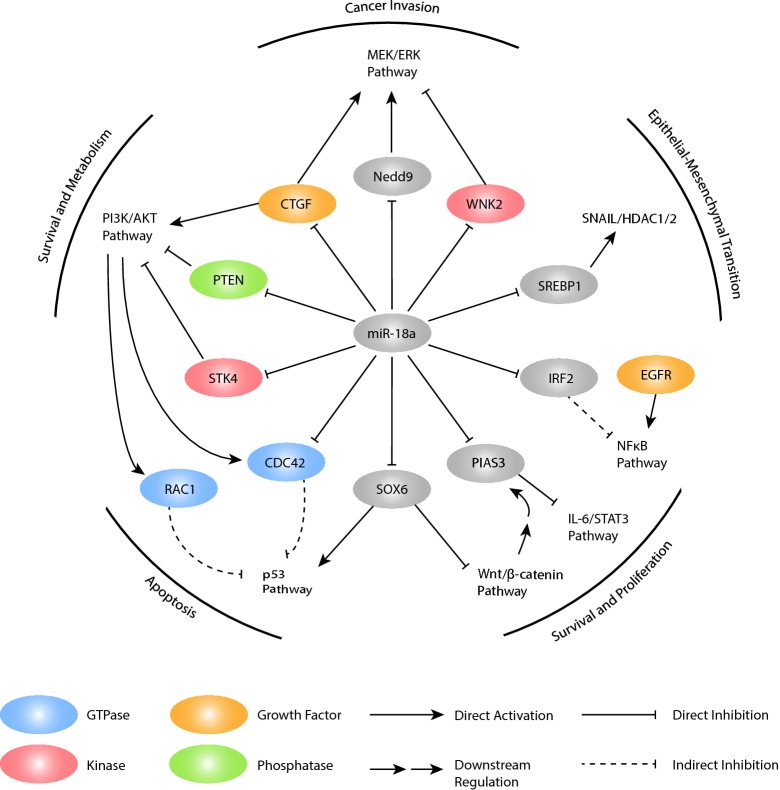
Signal transduction of miR-18a, with direct targets and associated pathways. The mechanisms of miR-18a regulation are characterized by direct activation or inhibition of the factors involved in signaling pathways that either promote or suppress cancer progression

A study of BC investigated the contributions of miR-18a to tumor progression and the regulatory mechanisms leading to miR-18a expression [69]. Among the downregulated members of the miR-17-92 cluster, miR-18a exhibited the most significantly decreased expression in highly lung-metastatic sublines derived from parental BC cells. Via microarray and bioinformatic approaches, this study verified that the 3′-UTR of sterol regulatory element-binding transcription protein 1 (SREBP1) is a target of miR-18a. Previous studies have shown that BC patients with higher SREBP1 expression had worse survival outcomes than those with lower SREBP1 expression, suggesting SREBP1 as a prognostic indicator in BC [70, 71]. In this study, SREBP1 was found to promote BC cell migration and invasion both in vitro and in vivo.

Furthermore, SREBP1 is significantly associated with EMT through E-cadherin repression, and this process is augmented by recruitment of the SNAIL/HDAC1/2 complex by SREBP1. Snail is a key EMT-related transcription factor that mediates E-cadherin repression by recruiting corepressors such as HDAC1/2 [72]. Upregulation of E-cadherin by SREBP1 knockdown is deacetylation-dependent. An article published this year also supported the hypothesis that SREBP1 drives BC cell invasion by activating cytoskeletal reprogramming. Moreover, this study proposed that reduced expression of miR-18a leads to SREBP1 overexpression, SNAIL/HDAC1/2 complex formation, E-cadherin inhibition, and eventually to EMT induction in BC cells. However, whether these components interact directly or indirectly remains unknown. The study revealed that miR-18a inhibited the progression and lung metastasis of BC by directly targeting SREBP1, which forms a corepressor complex with Snail and HDAC1/2 to modulate EMT, thus suggesting promising drug targets for novel therapeutic strategies for invasive and metastatic BC.

Interestingly, other studies have reported that E-cadherin suppression by the SNAIL/HDAC1/2 complex regulates metastasis in pancreatic cancer (PC) [73] and CRC [74], consistent with the hypothesis proposed in the BC study. Another study revealed that miR-18a inhibits the proliferation of adult pancreatic progenitor cells by suppressing the activity of the proliferation-related PI3K/AKT and ERK signaling pathways [75]. The SNAIL/HDAC1/2 complex does not regulate this process but instead inhibits the expression of connective tissue growth factor (CTGF); neural precursor cell, developmentally downregulated 9 (Nedd9); cyclin-dependent kinase 19 (CDK19); and insulin-like growth factor 1 (IGF1). *Cyclin D* genes are downstream of ERK and AKT [76], indicating that miR-18a suppresses cell cycle progression by downregulating *cyclin D* expression. However, the study that suggested SREBP1 and c-myc promote EMT in CRC did not mention miR-18a. Previously, we discussed that miR-18a suppresses CRC by inhibiting CDC42 expression. However, whether miR-18a promotes CRC metastasis by regulating SREBP1 requires further investigation.

#### Opposing effects of miR-18a have been observed in cancers of the same organ at different developmental stages and of various histologic subtypes, suggesting that the dual function of miR-18a may be attributed to the fundamental mechanisms of tumorigenesis

The preceding text indicates that the oncogenic and tumor-suppressive roles of miR-18a have been observed in cancers from totally different organs or tissues. Interestingly, differential miRNA expression is also described in various histologic subtypes and stages of human cancers of the same organ. A study of lung cancers proposed a model of lung adenocarcinoma oncogenesis from noninvasive precursor lesions through multiple steps, including atypical adenomatous hyperplasia (AAH) and bronchioloalveolar carcinoma (BAC). Across this AAH-BAC-adenocarcinoma development sequence, immunohistochemical analysis showed high Dicer expression in AAH and even higher Dicer expression in BAC but a decrease in Dicer expression in lung adenocarcinomas with stromal invasion. As discussed previously, Dicer is a key effector protein for miRNA biogenesis and function. The increase in Dicer in early precursor lesions compared to the decrease in late invasive adenocarcinomas suggests that the miRNA expression profile differs across different stages of lung cancer development. Furthermore, expanded immunohistochemical studies showed higher Dicer expression levels in squamous cell carcinoma (SCC) of the lung than in invasive adenocarcinoma, suggesting that miRNA stoichiometry is dependent on histologic subtype in lung cancer [77].

In summary, studies in different tumor types have revealed the oncogenic and tumor-suppressive roles of miR-18a. The unique dual function of miR-18a has also been observed in cancers of the same organ or tissue at different developmental stages or of various subtypes. Considering that cancers of the same organ can develop from completely different genetic contexts and cell origins, the dual function of miR-18a may be attributed to the fundamental differences in tumorigenic mechanisms.

However, the sample sizes of the studies focusing on miR-18a are relatively small, and further studies are required. Despite these study limitations, miR-18a has been confirmed to play a regulatory role in human cancer development and may be a target of future cancer treatment. Considering the dual functional role of miR-18a, the design of therapeutic regimens presents a challenge. We address the clinical potential of miR-18a in the following section.

### MiR-18a in clinical applications

**Fig. 4:**
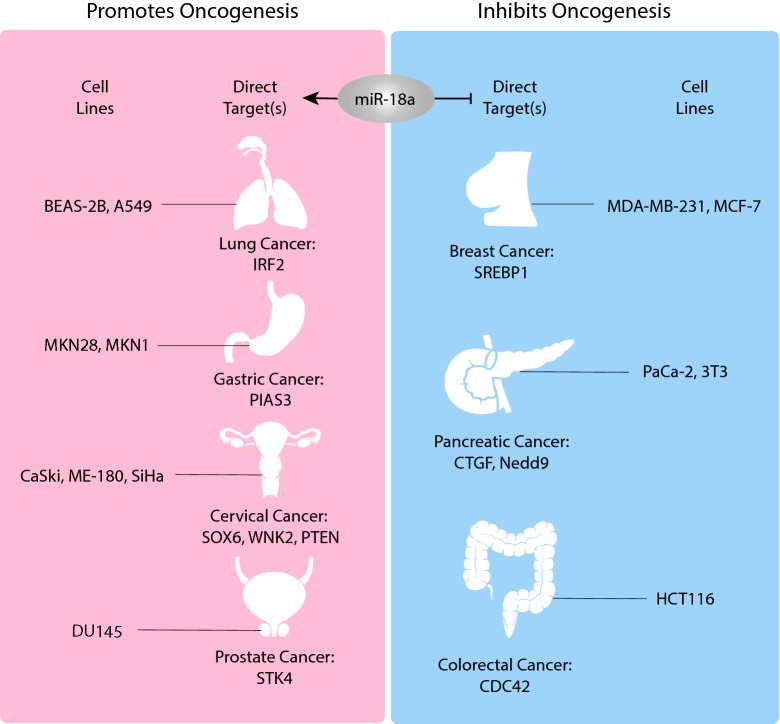
The dual functional role of miR-18a in different human cancers. miR-18a is overexpressed in various human cancer cell lines and promotes oncogenesis in lung, gastric, cervical, and prostate cancers but inhibits oncogenesis in breast, pancreatic, and colorectal cancers. This dual function is observed both in different human cancer types and in cancers of the same organ or tissue

Because miR-18a is closely related to oncogenesis, many studies have been eager to offer clinical translations in order to facilitate cancer screening, diagnosis, and treatment. MiR-18a exerts its dual functional role through overexpression and targeting the factors involved in pathways that either promote or inhibit oncogenesis (Fig. 4). Based on this observation, clinical applications of miR-18a as a biomarker are more well studied and understood than those of miR-18a as a therapeutic drug target.

Recently, circulating miRNAs contained in secretory vesicles have been reported to be stably detectable in human plasma and serum [78]. They are resistant to endogenous ribonuclease activity because they are packed into apoptotic bodies and exosomes [79]. These miRNA-containing secretory vesicles function as intracellular transmitters, suggesting that circulating miRNAs could be novel biomarkers for human cancer screening. Furthermore, most miRNAs, including miR-18a, are stable in plasma via binding the AGO-2 protein [80].

In this section, we summarize the expression of miR-18a in various types of cancer tissues and in peripheral blood (Table 2) according to previously published articles, showing the presence of circulating miR-18a and its potential use as a diagnostic marker for NSCLC [81], GC [82], CRC [83], and BC [84]. These reports provide evidence that plasma miR-18a may contribute to next-generation cancer screening via noninvasive liquid biopsy by functioning as a sensitive cancer biomarker. We also summarize findings identifying miR-18a as a chemoradiotherapy sensitizer, which suggest that miR-18a could be used to increase radiosensitivity and reverse radioresistance [85]. Finally, we introduce the delivery of anti-miRNA oligonucleotides by lipid nanoparticles (LNPs) as a new drug design approach [86]. When the molecular mechanism by which miR-18a regulates cancer development is thoroughly understood, LNPs could serve as a novel approach for the design of miR-18a-targeted therapy.

**Table 2:** The dual function of miR-18a in human cancers and miR-18a clinical applications

Cancer type	Expression status in cancer tissues and cell lines	Level in peripheral blood (plasma/serum)	Effect on oncogenesis	Potential clinical applications	Sample	Result	References
Non-small-cell lung cancer	Overexpressed	Increased	Promotes	Diagnostic marker	Peripheral blood miR-18a	AUC = 0.76 (95% CI 0.67–0.86)	[54, 81]
				Chemoradiotherapy sensitizer	–	–	[85]
Cervical cancer	Overexpressed	Unknown	Promotes	Unknown	Unknown	Unknown	[56]
Prostate cancer	Overexpressed	Unknown	Promotes	Unknown	Unknown	Unknown	[58]
Gastric cancer	Overexpressed	Unknown	Promotes	Diagnostic marker	Plasma miR-18a	AUC = 0.8059	[61, 82]
Colorectal cancer	Overexpressed	Increased	Inhibits	Diagnostic marker	Serum miR-18a	2.229-fold change (p = 0.038)	[68, 83]
Breast cancer	Overexpressed	Increased	Inhibits	Diagnostic marker	Serum miR-18a	19.00% change (p = 0.04)	[69, 84]
Pancreatic cancer	Overexpressed	Increased	Inhibits	Unknown	Unknown	Unknown	[75]
Hepatocellular carcinoma	Overexpressed	Increased	Unknown	Drug target	Unknown	Unknown	[86]

### MiR-18a as a biomarker for NSCLC

Recent studies in lung cancer have shown that miR-18a has good potential as a diagnostic biomarker for NSCLC. One study first analyzed a panel of miRNAs in peripheral blood samples from 86 NSCLC patients and 24 healthy donors and found that the expression levels of miR-328, miR-18a, miR-339, and miR-140 were significantly higher (p < 0.0001, p = 0.001, p = 0.001, and p = 0.021, respectively) in NSCLC patients than in healthy donors. Among the biomarker candidates, miR-18a showed good diagnostic accuracy in differentiating between patients with early NSCLC and healthy donors (area under the receiver operating characteristic curve (AUC ROC), 0.76; 95% CI 0.67–0.86) [81]. This noninvasive peripheral blood test of miR-18a levels could be implemented to screen for individuals at high risk of NSCLC with suspicious nodules detected by CT scans, avoiding the need for invasive biopsies.

### MiR-18a as a biomarker for GC

MiR-18a could also be a novel plasma biomarker in GC patients. One study evaluated the plasma level of miR-18a by comparing 104 GC patients and 65 healthy volunteers and found that plasma miR-18a concentrations were significantly higher in GC patients than in healthy controls (p < 0.0001). The AUC value was 0.8059, indicating that circulating miR-18a could be a useful biomarker for GC screening. This study also showed that plasma miR-18a concentrations were lower in postoperative GC samples than in preoperative samples (p = 0.0002), indicating that plasma miR-18a levels could also be used to monitor tumor dynamics [82]. One caveat regarding plasma miRNA assays is that circulating miRNAs can also originate from peripheral blood cells. To overcome this issue, the study evaluated the correlation between plasma miR-18a levels and peripheral blood cells and confirmed no significant correlations. Therefore, plasma miR-18a is a good diagnostic marker for GC.

### MiR-18a as a biomarker for other cancers

In a study of miRNA expression profiles in stage III CRC, peripheral blood samples were collected from 30 CRC patients and 26 healthy volunteers. The serum level of miR-18a was significantly higher in CRC patients than in healthy individuals (p < 0.05); specifically, the level of miR-18a exhibited a mean fold change of 2.229 (p = 0.038) between the serum samples of CRC patients and those of healthy individuals [83]. Another study on BC used serum data collected from the prospective cohort “Sister Study”, which included 205 BC patients and 205 controls, and found overexpression (19%) of serum miR-18a in BC patients compared to the controls [84].

Although these two studies indicated that higher circulating miR-18a levels in CRC and BC patients than in the corresponding healthy individuals, the changes of twofold and 19% were considered small compared to the changes in other biomarkers. Previously, we discussed the dual functional role of miR-18a in cancer development and suggested that miR-18a plays an inhibitory role in CRC and BC. This dual role may be the reason that the change in the level of circulating miR-18a is nonsignificant in these cancers. The correlation of the inhibitory mechanism of miR-18a with the observed changes in the circulating miR-18a level is unclear; thus, whether miR-18a is a good biomarker for CRC and BC remains to be further studied.

### MiR-18a as a chemoradiotherapy sensitizer

Regarding cancer treatment, one study showed that the expression of miR-18a in NSCLC tumor tissues was correlated with the therapeutic effects of radiotherapy. However, few reports have investigated the mechanism by which plasma miR-18a contributes to increased radiosensitivity in NSCLC patients. To investigate the role of miR-18a in NSCLC treatment, a group of researchers enrolled patients with stage IIIa-IIIb, unresectable lung cancer who were either obtaining a benefit from chemoradiotherapy or exhibited resistance [85]. Consistent with previous studies, the plasma miR-18a levels were significantly higher in the radiosensitive group than in the radioresistant group. Moreover, the objective response rate (ORR), which is defined as the proportion of patients attaining a complete response (CR) or partial response (PR), was significantly higher in the miR-18a-high group. A cutoff value of a relative miRNA expression level of > 2.28 was generated from the ROC curve, and the sensitivity and specificity of plasma miR-18a for predicting radiosensitivity were 86.7% and 94.9%, respectively.

Additionally, this research group indicated that miR-18a potentially increases radiosensitivity by targeting ataxia telangiectasia mutated (ATM) and hypoxia-inducible factor 1 alpha (HIF-1α) in lung cancer cells and CD133^+^ stem cells. A similar mechanism found in BC [87, 88], CC [89], CRC [90], GC [91] and meningiomas [92], supporting the hypothesis that miR-18a enhances radiosensitivity by downregulating both ATM and HIF-1α. Collectively, these findings indicate that miR-18a could be a promising radiosensitizer and could improve the benefit of radiotherapy for patients with a broad range of human cancers. Because most patients undergoing chemoradiotherapy develop radioresistance, miR-18a could also be introduced to reverse resistance.

### MiR-18a as a potential target for drug design

Although many basic studies have revealed that miR-18a is a promising biomarker in cancer screening, due to its dual functional role in cancer development, few studies have indicated that miR-18a is a potent drug target. The delivery of anti-miRNA oligonucleotides by LNPs has been strongly expected to demonstrate promising pharmacological effects in targeted cancer therapy. Indeed, one recent study claimed to provide proof-of-concept for targeting multiple members of the miR-17 family, including miR-18a, in hepatocellular carcinoma (HCC) treatment [86]. In this study, the research group validated the upregulation of miR-18a in HCC tumors and cell lines. Furthermore, systemic LNP-based delivery of an anti-miR-17 family oligonucleotide demonstrated its effect on inhibiting orthotopic Hep3B tumor growth in vivo. Although the mechanism remains unknown, pathway analysis indicated that the direct gene targets are associated mainly with the TGF-β pathway. This concept needs further investigation, because miR-18a has never been reported to be directly related to HCC via the TGF-β pathway. However, the correlation between the induction of miR-17 family member expression and the suppression of Hep3B tumor growth was demonstrated, thus offering insights into designing LNP-delivering oligonucleotides targeting miR-18a for cancer treatment. These studies provide a new method of drug design and a powerful application of miRNAs in cancer treatment.

## Conclusions

MiR-18a is unique due to a conserved sequence responsible for multistep RBP-dependent biogenesis at the Drosha-mediated level and is also involved in essential biological and pathological processes, especially those mediating human cancer developments. In lung, gastric, cervical, and prostate cancers, miR-18a was shown to promote oncogenesis. In contrast, in breast, pancreatic and colorectal cancers, miR-18a inhibited malignant progression. The dual functional role of miR-18a is supported by the identification of proposed signaling pathways involving cell proliferation, migration, invasion, apoptosis, and EMT. Furthermore, cancers of the same organ at different stages or of various subtypes exhibit differential miRNA expression, suggesting that the dual function of miR-18a is attributed to changes in fundamental mechanisms of tumorigenesis, not the cancer type. Circulating miR-18a is a potential biomarker that could be used in cancer screening and diagnosis. MiR-18a has also shown value as a chemoradiotherapy sensitizer and as a drug target via LNP-mediated delivery. Considering the dual function of miR-18a in cancer development, further investigations are needed to design therapeutic regimens for cancer treatment.

## Data Availability

Not applicable.

## Abbreviations

AGO: Argonaute
CDC42: cell division control protein 42
CPSF3: cleavage and polyadenylation specificity factor subunit 3
CTGF: connective tissue growth factor
DGCR8: DiGeorge syndrome chromosomal (or critical) region 8
EMT: epithelial–mesenchymal transition
HSC70: heat shock cognate 70
HSP90: heat shock protein 90
IRF2: interferon regulatory factor 2
ISY1: ISY1 splicing factor homolog
LNP: lipid nanoparticle
MYOD1: myoblast determination protein 1
Nedd9: neural precursor cell expressed, developmentally downregulated 9
PARP: poly-(ADP-ribose) polymerase
PIAS3: protein inhibitor of activated signal transducer and activator of transcription 3 (STAT3)
PTEN: phosphatase and tensin homolog
RISC: RNA-induced silencing complex
SOX6: sex-determining region Y (SRY)-box 6
SREBP1: sterol regulatory element-binding transcription protein 1
STK4: serine/threonine-protein kinase 4
TRBP: transactivation response (TAR) RNA-binding protein
WNK2: WNK lysine deficient protein kinase 2
